# Modeling pathogenic B cell function in experimental autoimmune encephalomyelitis

**DOI:** 10.1093/jimmun/vkag163

**Published:** 2026-07-11

**Authors:** Mohan Kumar, Connor R Wilhelm, Alexander W Boyden

**Affiliations:** Department of Pathology, University of Iowa, Iowa City, IA, United States; Department of Pathology, University of Iowa, Iowa City, IA, United States; Experimental Pathology PhD Graduate Program, Carver College of Medicine, University of Iowa, Iowa City, IA, United States; Department of Pathology, University of Iowa, Iowa City, IA, United States; Experimental Pathology PhD Graduate Program, Carver College of Medicine, University of Iowa, Iowa City, IA, United States

**Keywords:** autoimmunity, B cells, EAE/MS

## Abstract

Success of B cell depletion therapy in multiple sclerosis (MS), an immune-mediated demyelinating disease of the central nervous system, places B cells and their functions center stage in efforts to fully understand pathogenesis. Historically, MS animal models of experimental autoimmune encephalomyelitis have featured B cell–independent disease driven by CD4 T cells activated against central nervous system myelin protein epitopes and have provided many insights into autoreactive T cell biology in the context of neuroinflammation and demyelination. Meanwhile, B cells’ growing significance in MS necessitated the development of B cell–dependent experimental autoimmune encephalomyelitis models, which have allowed for critical exploration into pathogenic B cell functions during immune-mediated demyelinating disease, including cytokine and antibody production, as well as antigen presentation to autoreactive CD4 T cells. These advances represent an important arena for investigating MS pathogenesis. In this review, we summarize the various approaches taken to engage pathogenic B cells in animal models of MS.

## Introduction

Multiple sclerosis (MS) is an immune-mediated demyelinating disease of the central nervous system (CNS) that affects nearly 3 million individuals worldwide.^1^ MS is characterized by the infiltration of immune cells into the CNS, which then orchestrate a pathogenic response targeted against the myelin sheath.^2^ Myelin destruction disrupts saltatory conduction and thus neuronal signaling, resulting in a variety of motor, sensory, and cognitive deficits. While its exact etiology is unknown, the risk of developing MS increases significantly if a parent has been diagnosed, and more if a sibling has been diagnosed, and disease risk doubles if a monozygotic twin has been diagnosed, emphasizing the genetic components involved.^3^ Indeed, genetic contributions to MS development risk are estimated to be 50%.^4^ Beyond this, environmental factors including viral infection are thought to influence peripheral immune cell activation and contribute to disease initiation and progression. It has been reported that individuals are 32 times more likely to develop MS if having first been infected by Epstein-Barr virus (EBV),^5^ although possible mechanisms are still being investigated. Despite the development of numerous disease-modifying therapies aimed at treating symptoms, MS remains incurable, and its global prevalence continues to rise.^6^

MS is thought to be primarily driven by autoreactive CD4 T cells, in part because the strongest genetic risk factors for MS are certain polymorphisms in HLA class II molecules and because CD4 T cells are sufficient to transfer disease in the animal model of MS, experimental autoimmune encephalomyelitis (EAE).^7–9^ Accordingly, much research has focused on characterizing pathogenic CD4 T cells during disease.^10^ A growing literature on CD8 T cells has also demonstrated these cells to play both pathogenic^11^ and regulatory^12^ roles in MS and EAE.

Evidence that B cells contribute to MS disease progression is suggested by the presence of oligoclonal immunoglobulin bands (OCBs) in the cerebrospinal fluid of over 95% of MS patients.^13^ These OCBs, the presence of which is used as a diagnostic criterion for MS, are produced by antigen-experienced, clonally expanded B cells, indicating local B cell activation. Despite the prominence of OCBs in MS, the antigen specificity of the antibodies (Abs) within OCBs remains unclear and is being actively investigated. Evidence identifies OCBs target certain viruses and myelin proteins in MS but may ultimately arise due to CNS damage and are therefore specific for cellular debris.^14–16^ A pathogenic role for serum Abs is supported by the therapeutic benefits of plasma exchange in a subset of MS patients experiencing severe relapses.^17^ Ultimately, clear evidence for the pathogenic role of B cells in MS is emphasized by the success of B cell depletion therapy (BCDT) in limiting relapses and slowing disease progression, becoming a first-line treatment option for many MS patients.^18^ While this suggests that B cells act pathogenically in MS, the pathogenic mechanisms disrupted by this type of therapy remain unclear. BCDT involves the monoclonal Ab (mAb) targeting of a pan B cell surface protein, CD20, which is downregulated on Ab-secreting plasma cells.^19^ It has been reported that total serum Ig levels, cerebrospinal fluid Ig levels, and OCB numbers do not change shortly after treatment,^20^ suggesting that B cells may utilize mechanisms beyond Ab production to promote disease. Indeed, B cells are potent antigen-presenting cells (APCs) and secrete proinflammatory cytokines, which may influence the activation and pathogenic character of autoreactive CD4 T cells.

Regulatory B cells have also been reported in MS and EAE to secrete anti-inflammatory IL-10, while plasma cells secrete anti-inflammatory IL-35.^21^^,^^22^ In MS patients, an IL-10 secretion defect has been observed in B cells, which may contribute to disease.^23^ Ultimately, heterogeneity inherent in B cell populations suggests that anti-CD20 BCDT is not only depleting pathogenic cells, but also potentially eliminating regulatory cells, thus highlighting the need for better characterization of B cell functions and phenotypes in vivo.

EAE is the most common animal model of MS and is a valuable tool for researching disease pathogenesis and regulation, particularly for the T cell immunology involved. Typically a spinal cord disease, EAE is characterized by ascending hind limb paralysis and approximates several key pathogenic features of MS including CNS inflammation, demyelination, axonal loss, and gliosis.^24^ EAE is actively induced most often through immunization with short, immunodominant peptide sequences from proteins expressed in the CNS myelin sheath, as well as whole protein preparations, emulsified with Complete Freund’s Adjuvant. These include myelin basic protein (MBP), myelin oligodendrocyte glycoprotein (MOG), and myelin proteolipid protein (PLP), among others. Notably, the mouse strain can determine if disease is monophasic (C57BL/6) or relapsing-remitting (SJL). Additionally, certain strain/peptide combinations require additional administration of pertussis toxin to weaken the blood brain barrier and lead to full disease induction.^25^ In addition to active immunization, spontaneous models of EAE have been developed where adaptive immune cells are engineered to express receptors that recognize specific myelin sequences.^26^ Finally, passive EAE involves the adoptive transfer of myelin-reactive CD4 T cells from diseased donor mice into healthy recipients, which is sufficient to transfer neuroinflammation, demyelination, and paralysis in the absence of Complete Freund’s Adjuvant.^27^ Despite the variety of disease induction methods, EAE literature is dominated by models that do not require pathogenic B cells. Given the growing need to fully understand B cells’ role in MS, researchers have continued to develop active induction and immune receptor transgenic models for investigating B cell–dependent demyelinating disease. This review summarizes the approaches taken to engage pathogenic B cells in animal models of MS and are listed by category in Table 1.

**Table 1:** EAE models that engage significant B cell functionality.

	Animal	Description of disease	Author, year
**Active induction models**			
**Guinea pig spinal cord homogenate, guinea pig MBP**	Lewis rats	B cell–dependent EAE. Antibody-dependent EAE. No change to T cell function without B cells.	Willenborg, 1983^28^; Willenborg, 1986^29^
**Mouse spinal cord homogenate**	Biozzi mice	Secondary progressive-like disease. CNS B cell recruitment at chronic phase of EAE. Anti-MOG antibodies detected in serum. BTK inhibition reduced EAE severity and demyelination.	Nishri, 2021^30^; Evonuk, 2023^31^
**rhMOG**	C57BL/6 mice	B cell–dependent EAE. Antibody-dependent EAE. B cell MHC class II expression required for EAE. B cell IL-6 production promotes EAE. Decreased proinflammatory CD4 T cells without B cells.	Molnarfi, 2013^32^; Lyons, 1999^33^; Lyons, 2008^34^; Lyons, 2002^35^; Monson, 2011^36^
**rhMOG**	HLA-DR4 transgenic mice	MOG_97–108_ was processed and presented by human B cells to CD4 T cells from humanized HLA-DR4 (DRB1*0401) mice.	Forsthuber 2001^37^
**rhMOG**	Marmoset	B cell–dependent EAE. Without B cells, decreased inflammatory cytokine–producing T cells and reduced serum anti-MOG antibodies.	Kap, 2010^38^
**Human MOG_34–56_**	Marmoset	B cell–dependent EAE. No changes to T cell function without B cells.	Jagessar, 2012^39^
**rHuMOG**	DA rats	Conformational anti-MOG antibodies present in the serum.	De Graaf, 2012^40^
**rrMOG_1–125_**	C57BL/6 mice	B cell–independent EAE. Severe disease induction after suboptimal immunization requires both 2D2 T cell and IgH^MOG^ B cell transfers.	Flach, 2016^41^; Faust, 2024^42^; Oliver, 2003^43^
**rrMOG_1–125_**	C3HeB/Fej mice	B cell depletion reduces incidence (but not severity) of EAE. Decreased inflammatory cytokine–producing T cells after B cell depletion. Decreased T cell retention to CNS with B cell depletion.	Stromnes, 2008^44^; Pierson, 2014^45^
**rrMOG_1–125_**	Lewis rats	Resistant to rrMOG_1–125_ EAE. Magnitude of EAE and anti-MOG antibody response dependent on MHC haplotype.	Weissert, 1998^46^; Weissert, 2001^47^
**rrMOG_1–125_**	Marmoset	Anti-MOG antibodies generated. Pathogenic IgG can increase EAE severity and demyelination in recipients with suboptimal EAE.	Genain, 1995^48^
**rmMOG_1–117_**	C57BL/6 mice	B cell depletion reduces EAE development. Serum anti-MOG antibodies generated. BTK inhibition reduced disease severity.	Weber, 2010^49^; Torke, 2020^50^
**bMOG**	C57BL/6 mice	BCR-restricted (B1-8^+/+^Jκ^–/–^) mice do not develop EAE, while WT C57BL/6 mice do.	Whittaker Hawkins, 2017^51^
**MOGtag**	C57BL/6 mice	Similar disease severity in WT and B1-8 (BCR-restricted) mice. Germinal centers generated and cluster at meninges.	Dang, 2015^52^
**MP4**	C57BL/6 mice	B cell–dependent EAE. Antibody-dependent EAE.	Kuerten, 2011^53^; Breakell, 2020^54^; Elliott, 1996^55^
**Mouse MBP**	PL/J, BALB/c × SJL F1 mice	B cell–dependent EAE. Antibodies exacerbate disease. Potential pathogenic B cell APC function.	Myers, 1992^56^
**PLP_ECD_**	C57BL/6 mice	B cell–dependent EAE. Antibody-independent EAE. Susceptible to B cell depletion therapy. B cell antigen recognition and presentation required.	Boyden, 2020^57^; Wilhelm, 2023^58^
**Transgenic models**			
**IgH^MOG-mem^ mice (active with rhMOG)**	C57BL/6 mice	Active, single transgenic EAE. B cell antigen recognition–dependent EAE. Antibody-independent EAE.	Molnarfi, 2013^32^
**2D2 × IgH^MOG^ (spontaneous OSE)**	C57BL/6 mice	Spontaneous, double transgenic EAE. Requires both MOG-reactive B and CD4 T cells. Anti-MOG antibodies are generated but not required for disease.	Krishnamoorthy, 2006^59^; Häusler, 2015^60^; Molnarfi, 2013^32^
**TCR^1640^ transgenic mice (spontaneous RR-EAE)**	SJL mice	Spontaneous, single transgenic EAE. B cell–dependent EAE. Serum anti-MOG antibodies enhance EAE but are insufficient to cause disease on their own.	Pöllinger, 2009^61^; Salvador, 2022^62^
**IgH^MOG^ mice (active with rrMOG_1–125_)**	C57BL/6 mice	Active, single transgenic EAE. Accelerated disease compared with nontransgenic mice.	Litzenburger, 1998^63^
**IgH^MOG^ mice (active with PLP_139–154_)**	SJL mice	Active, single transgenic EAE. PLP-specific T cell transfer accelerates disease. Cooperation between B and T cells.	Litzenburger, 1998^63^
**IgH^MOG^ mice (active with MOG_35–55_)**	NOD mice	Active, single transgenic EAE. No difference in serum anti-MOG IgG versus nontransgenic mice. More CNS IL-6– and IL-23–producing B cells versus nontransgenic mice. Blocking IL-23 prevents EAE.	Fazazi, 2024^64^
**B^MHCII ×^ IgH^MOG^ mice (passive EAE from MOG_35–55_-immunized CD4 T cell donor)**	C57BL/6 mice	Passive, B cell–dependent, double transgenic EAE. CD4 T cells from MOG_35–55_–immunized donors transfer disease to mice with both antigen presentation restricted to B cells and a B cell population skewed to recognize MOG.	Parker Harp, 2015^65^; Archambault, 2013^66^

## Active induction models

Several animal species, such as mouse (the most common) and marmoset, have been utilized for active induction (injections) to understand potential pathogenic B cell involvement in EAE and, notably, have built upon important early work in rats in which pathogenic B cell function was indicated. Key findings from such models are presented subsequently and organized by induction methodology, as this heavily influences the B cell requirement. These include short myelin peptides that are largely B cell independent, as well as tissue homogenates and MOG/non-MOG reagents of varying B cell dependence.

### Short myelin peptide–induced EAE

Historically, active EAE has been induced using short immunodominant myelin peptide sequences from MOG (MOG_35–55_, MOG_92–106_), PLP (PLP_139–151_, PLP_178–191_), and MBP (MBP_84–104_, MBPAc_1–11_).^25^^,^^67^ Immunization with these epitopes induce B cell–independent EAE.^57^^,^^68^^,^^69^ For example, wild-type (WT) C57BL/6 (B6), B cell–deficient (µMT), and B cell–depleted (anti-CD20 mAb treated) mice all develop similar EAE when immunized with MOG_35–55_.^21^^,^^33^^,^^70^^,^^71^ Likewise, similar disease severity develops in both B cell–deficient µMT mice and anti-CD20–treated (B cell–depleted) B6 mice compared with B cell–sufficient counterparts when immunized with PLP_178–191_.^57^^,^^58^

Depleting B cells after MOG_35–55_–induced disease onset reduced disease severity in B6 mice and suggests that these cells act pathogenically at later stages of disease.^70^ B cell–produced IL-6 was observed to be a critical part of B cell–mediated pathogenicity in this model, as chimeric mice in which B cells alone lack the ability to produce IL-6 experienced less severe MOG_35–55_ EAE than WT control mice.^72^ Additionally, depleting B cells from these chimeric mice after disease onset showed no additional reduction in disease severity, whereas WT mice showed a reduction in symptoms. This suggests that the benefits of BCDT in this model arise, in part, from eliminating IL-6–producing B cells. A minor pathogenic Ab role was shown to be elicited in A.SW mice immunized with MOG_92–106_, in which serum MOG_92–106_–reactive Ab responses were associated with disease progression and Ab deposition linked to demyelinated lesions in the CNS.^73^ MBP_84–104_ has been explored sparsely when evaluating B cells in EAE, though one study found elevated levels of anti-MBP_84–104_ IgG Abs in MS patients compared with healthy control mice.^74^

Short PLP peptides including PLP_139–151_, PLP_178–191_, and more vary in their engagement of B cells during EAE. B6 mice show similar disease severity in both B cell–deficient µMT mice and anti-CD20–treated B cell–depleted mice compared with WT counterparts when immunized with PLP_178–191_.^57^^,^^58^ In SJL mice immunized with PLP_139–151_ to model relapsing-remitting disease, there was an increased number of B cells in the CNS, but a pathogenic role for these cells was not confirmed.^75^ In another study, it was found that PLP_139–151_–immunized SJL mice could produce anti-PLP_139–151_ IgG Abs, but it is unknown if these Abs were pathogenic.^76^ Finally, blockade of B7-1-mediated costimulation in acute PLP_139–151_–induced disease prevented further relapses, suggesting a pathogenic role for B7-1–mediated costimulation, which may involve B cells.^77^

Given the nature of B cell receptors (BCRs), groups have explored whether tissue homogenates, larger peptides, or recombinant proteins have the potential to better engage pathogenic B cells. Unlike processed short linear peptides presented on major histocompatibility complex (MHC) molecules, larger protein sequences exhibit a 3-dimensional conformation that may contain multiple epitopes capable of being bound by multiple BCRs. This supports BCR crosslinking, a process that contributes to robust B cell activation and receptor-mediated endocytosis. While small monovalent epitopes can activate B cells, this process is less efficient without BCR crosslinking.^78^ With these BCR engagement nuances in mind, approaches in the next few sections induce pathogenic B cell functions to a greater degree than small peptides.

### Spinal cord homogenate–induced EAE

The earliest evidence for pathogenic B cells in EAE came from the observation that anti-IgM treated, B cell–depleted Lewis rats failed to develop disease after immunization with whole guinea pig spinal cord homogenate or purified guinea pig MBP, compared with B cell–sufficient rats. These B cell–depleted rats also failed to develop histological signs of inflammation in the CNS and did not produce anti-MBP Abs.^28^ Disease was restored in these rats after receiving serum from B cell–sufficient, MBP-sensitized mice,^29^ indicating that B cell–derived anti-myelin Abs were necessary for disease upon active immunization with MBP. B cell depletion did not dramatically alter T cell function, as these rats rejected tissue allografts similarly to B cell–sufficient rats.^28^ CD4 T cells from B cell–depleted, MBP-sensitized rats were able to adoptively transfer disease to naive recipients,^29^ suggestive of indirect regulation of full T cell pathogenic potential by pathogenic B cells or Abs.

Immunizing Biozzi mice with spinal cord homogenate from Swiss Webster mice induces a nonresolving, secondary progressive-like disease that features demyelination and pathogenic B cell engagement.^30^^,^^79^ This disease is characterized by robust spinal cord T cell infiltration in the acute phase followed by B cell recruitment in the chronic phase, suggesting a dominant role for B cells in late stages of EAE. Supporting this idea, anti-MOG Ab reactivity was low in the acute phase and high in the chronic phase of disease.^30^ However, another study reported that B cell depletion does not reduce EAE severity in this model.^80^ Interestingly, Bruton’s tyrosine kinase (BTK) inhibitors, which disrupt B cell maturation without causing widespread depletion, reduced EAE severity in these mice when administered both before disease induction and during the chronic phase.^31^ Chronic-phase BTK inhibitor administration also reduced demyelination, myeloid cell infiltration, and microglia activation in the spinal cord. While spinal cord B cell numbers do not change, splenic B cell maturation was disrupted, suggesting that BTK inhibitors work by reducing pathogenic B cell function in this model.^31^ The large, natively folded protein structures in the studies described previously may contribute to more efficient B cell activation, while the short length of more common EAE-inducing peptides (like MOG_35–55_, PLP_139–151_, and PLP_178–191_) may not sufficiently engage B cells. However, peptide length does not always explain these dynamics, as the source of myelin sequence (rodent or human) can matter a great deal, which is the case for MOG protein–induced EAE models detailed in the next section.

### MOG protein–induced EAE

Few B cell–dependent active induction EAE models have been developed. Historically, the most utilized method involves immunizing B6 mice with recombinant human MOG (rhMOG).^33–35^ This peptide encompasses the extracellular domains, including the MOG_35–55_ sequence, of human MOG protein and induces robust B cell–dependent EAE. While severe inflammation, demyelination, and axonal loss were detected in the CNS of rhMOG-immunized B cell–sufficient mice, these pathologic hallmarks were dramatically reduced in mice lacking B cells.^33^ Critically, transferring B cells or serum from rhMOG-primed donors restored susceptibility to disease in rhMOG-immunized, B cell–deficient mice, highlighting autoreactive Abs as a key pathogenic component of disease.^35^ Despite not developing EAE, MOG_35–55_–primed T cells are detected in B cell–depleted rhMOG-immunized mice.^33^ Interestingly, transferring rhMOG serum at later stages of disease resulted in rapid increases in clinical score.^35^ Notably, these Abs were unable to transfer disease on their own,^35^ indicating that other pathogenic mechanisms are at play in this model. In addition to producing Abs, B cells presented antigen to CD4 T cells and secreted inflammatory cytokines, influencing T cell activation and polarization. Bone marrow chimeric mice with a deficiency in MHC class II restricted only to B cells were resistant to rhMOG EAE, indicating a role for B cell–mediated antigen presentation.^32^ In this study, the addition of anti-MOG Abs only partially restored EAE symptoms, demonstrating that, while pathogenic, autoantibodies were insufficient to compensate for a lack of B cell antigen presentation. Compared with control mice, fewer CD4 T cells from these mice expressed the inflammatory cytokines IFNγ and IL-17A, suggesting that B cells influence the pathogenic character of CD4 T cells. Notably, a similar amelioration of EAE and reduction in IL-17A–producing T cells was observed in rhMOG-immunized mice expressing both human CD20 and mouse CD20 (CD20dbtg) that were depleted of B cells using the humanized mouse mAb rituximab.^36^ The cytokine IL-6 is known to be produced by B cells and plays a role in polarizing naive CD4 T cells into IL-17A–producing Th17 cells.^81^ In mice in which B cell IL-6 production was eliminated using a cre/flox system, rhMOG-driven disease was significantly reduced compared with control mice, identifying B cell–provided cytokines as an important pathologic mechanism.^32^ Interestingly, a role for human B cells as presenters of rhMOG-derived peptides has been identified using CD4 T cells from humanized B6 mice. In humans, HLA-DR4 (DRB1*0401) is strongly associated with MS susceptibility. MOG_97–108_ is the immunodominant HLA-DR4 (DRB1*0401)–restricted T cell epitope of rhMOG-immunized mice.^37^ CD4 T cells from such mice following MOG_97–108_ immunization exhibited vigorous cytokine production when cocultured with human HLA-DR4 homozygous B cells after either MOG_97–108_ or rhMOG stimulation, demonstrating a role for human B cells as myelin antigen presenters.^37^ Taken together, rhMOG-driven EAE is B cell dependent and driven by both autoantibody generation and augmentation of CD4 T cells through B cell antigen presentation and cytokine production.

As in mice, rhMOG immunization also induces B cell–dependent EAE in marmosets.^38^ After immunization, treatment with an anti-CD20 mAb reduced clinical signs of disease. Additionally, B cell depletion prevented the development of CNS white matter lesions, as detected by magnetic resonance imaging. CD3+ T cells from the axillary lymph nodes of B cell–depleted marmosets produced less IFNγ, IL-17A, TNFα, and IL-6 than cells from their B cell–sufficient counterparts. Of note, B cell–depleted marmosets showed reduced serum anti-MOG Abs, although their role in this model is unclear. Interestingly, immunizing marmosets with the much shorter human MOG_34–56_ also induces B cell–dependent EAE, evidenced by reduced disease in B cell–depleted marmosets compared with B cell–sufficient ones.^39^ B cell–depleted human MOG_34–56_–immunized marmosets had less inflammation, demyelination, and lesions in the CNS. Despite the effectiveness of B cell depletion, significant differences in T cell inflammatory cytokines were not detected between B cell–sufficient and B cell–deficient marmosets in this study. B cell depletion did not alter anti-MOG_34–56_ IgM levels in serum, suggesting that the therapeutic benefits of B cell depletion in this model are Ab independent.^39^

Interestingly, Dark Agouti (DA) rats immunized with rhMOG sequences exhibit varying degrees of Ab response depending on the use of correctly folded protein. EAE fails in DA rats immunized with standard rhMOG, while immunizing with a version containing the native extracellular disulfide bond (rHuMOG) induced robust EAE and increased anti-MOG Abs present in the serum.^40^ T cell responses against the MOG_91-108_ epitope were also larger in rHuMOG-immunized DA rats, suggesting enhanced antigen processing/presentation in this model.

While human MOG sequences induce B cell–dependent EAE in several animal models, EAE induced with MOG proteins derived from rodents can be more nuanced. Mouse and rat MOG, while extremely similar, have differences in their effects on B cells.^42^^,^^43^ They each have been used in various studies including recombinant rat MOG_1–125_ (rrMOG_1–125_), recombinant mouse MOG_1–117_ (rmMOG_1–117_), and humanized rat MOG_1–125_. These will be discussed subsequently by specifying the species and peptide used in each study.

rrMOG_1–125_ induces disease in both B cell–sufficient and B cell–deficient mice, despite rrMOG_1–125_ being of comparable length to rhMOG.^43^ This demonstrates that sequence length does not fully explain why certain myelin derivatives induce B cell–dependent EAE, and others do not. Of note, a difference at amino acid position 42, which in rhMOG (human) contains a proline and in rrMOG (rodent) contains a serine, has high pathologic significance. Substituting a serine for proline at position 42 of rhMOG eliminates the B cell–dependent nature of this protein, indicating that small variations in the immunodominant sequence based on the source of MOG antigen can determine if B cells are required for disease.^82^ Notably, despite rhMOG inducing severe B cell–dependent EAE that requires proline in position 42, immunization with human MOG_35–55_, which contains a proline in this position, induced weak EAE.^43^

While rrMOG_1–125_–induced EAE is B cell independent, B cells are involved in this model when using suboptimal disease induction and myelin reactive immune cell transfers. T cells from mice with TCRs that recognize MOG_35–55_ (2D2) and B cells from mice that recognize MOG because they express an Ig heavy chain from the MOG-specific hybridoma 8.18-C5 (IgH^MOG^) were transferred into B6 mice immunized with a suboptimal dose of rrMOG_1–125_. While transferring 2D2 T cells alone after immunization induces weak EAE, transferring both 2D2 T cells and IgH^MOG^ B cells leads to more severe disease, indicating that IgH^MOG^ B cells enhance rrMOG_1–125_–driven EAE.^41^ Intravital two-photon microscopy revealed earlier and larger 2D2 T cell infiltration into the spinal cord when mice received both 2D2 T cells and IgH^MOG^ B cells than when they received 2D2 T cells alone, showing that IgH^MOG^ B cells promote T cell entry into the CNS. Despite these findings, 2D2 T cell priming was not changed by the presence of IgH^MOG^ B cells. Interestingly, if the cotransferred IgH^MOG^ B cells cannot produce Abs, they no longer accelerate disease induction, indicating that Abs play a major role in IgH^MOG^ B cell–mediated disease exacerbation. While this provides information regarding the importance of IgH^MOG^ Ab production during rrMOG_1–125_–driven disease, the role of rrMOG_1–125_ Abs has not been directly tested.^41^

While most EAE models feature inflammation primarily in the spinal cord, rrMOG_1–125_ immunization in C3HeB/Fej mice produces parenchymal lesions in both the brain and spinal cord, mirroring what is seen in MS.^44^ Interestingly, B cell–deficient C3HeB/Fej mice immunized with rrMOG_1–125_ develop EAE at significantly lower incidence than their B cell–sufficient counterparts, indicating a role for B cells in the initiation of disease.^45^ Despite decreased incidence, when disease does develop in B cell–deficient C3HeB/Fej mice, day of onset, severity, and brain infiltration were similar to B cell–sufficient mice, suggesting that B cells are less essential once EAE is initiated. B cells appear to influence T cell priming in this model, as significantly fewer splenocytes from B cell–deficient C3HeB/Fej mice produced IFNγ and IL-17A after being stimulated with MOG_97–114_. Notably, bulk splenocytes from rrMOG_1–125_–immunized donor mice restimulated with MOG_97–114_ transfer disease to B cell–sufficient recipients significantly more than B cell–deficient recipients. This suggests that B cells are not only necessary for the priming of T cells, but also continue to play a role at the effector stages of disease. Additionally, the number of donor CD4 T cells in the CNS, while similar at preclinical stages of disease, was dramatically lower in B cell–deficient recipients at later time points, suggesting that host B cells promote additional CD4 T cell recruitment and/or retention to the CNS in C3HeB/Fej mice. B cells were important in reactivating CD4 T cells in the CNS through antigen presentation, but the role of Abs was not tested in this model.

Standard Lewis rats are resistant to rrMOG_1–125_–driven EAE and show weak anti-MOG Ab responses. However, different MHC haplotypes in these rats influence disease susceptibility and magnitude of the B cell response.^46^ While rrMOG_1–125_–specific Ab responses are not sufficient to induce disease, complement deposition in CNS lesions suggests that anti-MOG Abs may exacerbate demyelination in this model.^47^

In marmosets, rrMOG_1–125_ immunization induces robust EAE with demyelinating lesions and antigen-specific T cell and Ab responses.^48^ Interestingly, while MBP-immunized marmosets develop mild EAE with no demyelination, transferring purified IgG from rrMOG_1–125_–immunized donors into MBP-immunized recipients exacerbates disease and yields demyelination, indicating a pathogenic role for Abs in this model. Importantly, the Ab transfer exacerbation effect required MBP immunization.^48^

Both B cell–sufficient and B cell–depleted B6 mice develop EAE when immunized with rmMOG_1–117_; however, B cell depletion after disease onset significantly reduced disease severity,^49^ suggesting that B cells play a role in supporting established disease. Postonset B cell depletion also reduced B cells in the CNS. IFNγ- and IL-17A–producing CD4 T cells were reduced in the periphery and to a greater extent in the CNS after postonset B cell depletion. Despite reduced EAE severity, postonset B cell depletion also reduced the number of Foxp3+ regulatory T cells in the CNS and periphery. Additionally, B cells seem to influence the inflammatory character of other APCs, as B cell depletion resulted in CD11b^+^ cells secreting the inflammatory cytokine TNFα. Anti-MOG Abs were generated after rmMOG_1–117_ immunization, and their serum titers were reduced when anti-CD20 was administered either before or after immunization. BTK inhibition in rmMOG_1–117_–immunized B6 mice reduced both clinical disease severity and histological signs of pathology.^50^ Notably, BTK inhibition did not reduce total circulating B cell numbers but did reduce B cell maturation, antigen presentation molecule expression, and ability to generate encephalitogenic T cells.

A humanized mouse MOG_1–125_ protein, referred to as bMOG, contains a S42P mutation to disrupt the immunodominant MOG_35–55_ epitope and was shown to engage pathogenic B cells. While WT B6 mice develop robust EAE after bMOG immunization, disease did not develop in BCR-restricted B1-8^+/+^Jκ^−/−^ mice, demonstrating the necessity of B cell antigen recognition in this model.^51^

Longer MOG sequences have thus been useful in investigating B cells in EAE, yet the expression and purification of properly folded antigens is a technically complex and time-consuming process. A novel reagent, termed MOGtag, was generated to address this and is composed of mouse MOG with a thioredoxin tag, which is expressed from easily grown bacteria and harvested using common lab reagents.^52^ MOGtag induces disease in both WT and B1-8 mice (mice with BCR specificity restricted to nitrophenol), indicating that MOGtag induces EAE regardless of BCR specificity.^52^ Germinal centers were generated and cluster at the meninges in this model, as in human MS.^83^

### Non–MOG protein–induced EAE

In addition to MOG, MS patients mount immune responses against other myelin proteins like MBP and PLP.^84–91^ While less well studied, derivatives from these myelin proteins can be used to induce EAE, some of which are B cell dependent. MP4 is a fusion protein that combines human MBP with the 3 hydrophilic loops of PLP.^55^ MP4-induced EAE is B cell–dependent in B6 mice, as B cell–deficient mice developed weaker EAE and less CNS pathology compared with WT control mice.^53^ Transferring serum from MP4-immunized WT mice into B cell–deficient, MP4-immunized mice restored susceptibility to disease, demonstrating a required pathogenic role for Abs in this model. Accordingly, significant Ab deposition was found in demyelinated plaques of MP4-immunized mice. A similar reduction in EAE severity and CNS pathology is observed in MP4-immunized CD20dbtg B6 mice that are depleted of B cells with the anti-human CD20 mAb obinutuzumab.^54^ In PL/J mice and BALB/c × SJL F1 mice immunized with mouse MBP, Abs exacerbated disease.^56^ EAE could not be induced in these mice if B cell depleted unless they were locally reconstituted with B cells prior to immunization, suggesting that B cells were acting as important APCs.^56^

PLP-reactive Ab levels are elevated in MS.^92^ PLP accounts for 50% of CNS myelin protein content in humans^93^ versus MOG’s 0.05%,^94^ and shares complete amino acid sequence homology between mice and humans,^92^^,^^95^ whereas MOG does not.^96^ A novel designed peptide spanning the extracellular domain sequences of PLP (PLP_ECD_) was recently shown by our group to induce robust EAE in B cell–sufficient but not B cell–deficient or B cell–depleted B6 mice.^57^^,^^58^ B cell–depleted mice exhibited less CNS inflammation and demyelination than B cell–sufficient mice. Anti-PLP_ECD_ Abs, while detected in PLP_ECD_-immunized mice, do not appear to be required, as mice containing mature B cells but lacking the ability to class switch or secrete Abs developed EAE upon PLP_ECD_ immunization.^57^^,^^58^ Depleting B cells after disease onset was shown to reverse the ongoing established disease course, indicating pathogenic B cell function throughout and demonstrating robust susceptibility to BCDT.^58^ Mechanistically, PLP_ECD_ EAE is dependent on B cell–mediated antigen presentation, as bone marrow chimeric mice in which MHC class II gene deficiency was restricted to the B cell compartment failed to develop disease compared with their gene-sufficient counterparts. B cell antigen recognition of PLP_ECD_ is required, as MD4 mice, featuring BCR specificity to hen egg lysozyme, failed to develop disease. Inguinal lymphocytes from B cell–depleted mice had fewer activated CD4 T cells and within the activated population had fewer IFNγ+ and IL-17A+ CD4s compared with B cell–sufficient mice. Additionally, CD4 T cells from B cell–depleted mice proliferated less than those from B cell–sufficient mice. These results indicated that B cells not only increase the number of activated CD4 T cells, but also enhance their pathogenic character as well. Accordingly, the inability of CD4 T cells from B cell–depleted, PLP_ECD_-immunized mice to transfer disease emphasizes the impact that B cells have over the pathogenic potential of CD4 T cells in this model.^58^

## Transgenic models

In addition to active immunization, a variety of transgenic models of EAE have been developed that feature pathogenic B cells. Spontaneous EAE occurs in mice with immune receptors engineered to recognize protein sequences within the myelin sheath. Opticospinal EAE (OSE) is a double transgenic, spontaneous model of EAE in B6 mice in which host CD4 T cells express TCRs that recognize MOG_35–55_ (2D2) and host B cells express an Ig heavy chain from the MOG-specific hybridoma 8.18-C5 (IgH^MOG^).^59^^,^^60^ Notably, only around 4% of single transgenic 2D2 mice develop classic EAE spontaneously,^97^ indicating that the MOG-recognizing B cells are important for robust and consistent EAE development in this model. OSE mice developed CNS lesions primarily in the optic nerve and spinal cord. These lesions showed significant inflammation and demyelination and were dominated by macrophages and CD4 T cells. These CNS CD4 T cells appeared to be activated, as they expressed CD69 and CD25. Real-time PCR of OSE spinal cords revealed significantly higher expression of IFNγ and TNFα compared with WT. Notably, OSE mice had significantly higher anti-MOG Ab titers than single transgenic IgH^MOG^ mice. The inability of single transgenic mice to induce high incidence disease suggests that B cells and T cells cooperate in the OSE model, with B cells enhancing T cell localization and activation and T cells increasing anti-MOG Ab production. To test the necessity of Abs, a similar double transgenic model was generated with identical TCR and BCR specificity but with B cells that could not secrete Abs. These IgH^MOG-mem^ × 2D2 mice developed spontaneous EAE and developed ectopic lymphoid-like follicles in their meninges.^32^ This indicates that while both B and T cells specific for MOG are needed for spontaneous OSE disease development, Abs may not be required.

Spontaneous, relapsing remitting EAE was developed in single transgenic SJL mice expressing TCRs that recognize rat/mouse MOG_92–106_ (TCR^1640^),^61^ although others found that this model results in a monophasic disease.^62^ Depleting B cells from TCR^1640^ mice prevented EAE development and serum anti-MOG IgG1 generation.^61^ Disease was associated with CNS inflammatory infiltrate and demyelination. Th1 and Th17 CD4 T cells were located within the CNS of sick mice, and real-time polymerase chain reaction of the CNS identified high levels of IFNγ and IL-17A, suggesting that the presence of B cells is required for the generation or localization of inflammatory CD4 T cells. The anti-MOG Abs generated from TCR^1640^ mice are pathogenic, as sera from diseased mice enhance EAE when transferred into PLP_139–151_–immunized mice or naive 2D2 mice.^61^^,^^62^ Despite the ability of such Abs to enhance disease, they were insufficient on their own, as indicated by their production in TCR^1640^ mice in which disease failed to develop.^62^ Of note, MOG tetramer–positive B cells significantly increase in both the CNS and cervical lymph nodes^62^ after disease onset.

While not spontaneous, other models engage pathogenic B cells through genetic manipulation coupled with active immunization or adoptive transfer of CD4 T cells. The previously mentioned IgH^MOG-mem^ mice, which have B cells that recognize MOG but cannot secrete Abs, do not develop spontaneous disease on their own but are susceptible to rhMOG-induced disease.^32^ Notably, in the absence of Abs, if BCR specificity were to an irrelevant peptide, rhMOG-induced disease did not develop. Spontaneous EAE did not develop in IgH^MOG^ mice when backcrossed onto the SJL background.^63^ However, SJL IgH^MOG^ mice immunized with PLP_139–154_ experienced disease development, CNS inflammation, and demyelination earlier than WT SJL mice. Similar increased disease incidence was seen in B6 IgH^MOG^ mice immunized with rrMOG_1–125_. Accelerated disease was also seen in SJL IgH^MOG^ mice after adoptive transfer of PLP-specific T cells generated from mice immunized with PLP_139–154_ and PLP_130–151_.^63^ Interestingly, this disease acceleration occurred despite genetic predisposition to MOG reactivity and having been challenged with a PLP antigen. The authors argue that this suggests that there may be no antigen-dependent cooperation between pathogenic B cells and T cells in this model. Rather, they argue that Abs likely exacerbate the immune response initiated by T cells, independent of antigen specificity.

Following MOG_35–55_ immunization, NOD IgH^MOG^ mice develop significantly more severe EAE, CNS immune cell infiltration, and CNS demyelination than WT NOD mice, indicating that B cells promote disease exacerbation in this model.^64^ Interestingly, there was no difference in serum anti-MOG IgG between WT and IgH^MOG^ NOD mice, de-emphasizing the role of pathogenic Abs. In the CNS of NOD IgH^MOG^ mice, a greater proportion of B cells produced IL-6 and a greater proportion of CD4 T cells produced IL-17A compared with WT NOD mice. NOD.Scid mice that receive both WT NOD B cells and MOG_35–55_–specific Th17 CD4 T cells develop moderate EAE; however, this disease was enhanced when B cells were sourced from IgH^MOG^ NOD mice. Notably, CNS B cells from MOG_35–55_–immunized, NOD IgH^MOG^ mice produced more of the Th17-stabilizing factor IL-23 than CNS B cells from WT NOD mice. IL-23 was shown to be required for MOG_35–55_–driven disease in IgH^MOG^ mice, as blocking IL-23 prevented EAE development.^64^ Together, this suggests that B cells enhance disease in IgH^MOG^ NOD mice by supporting CNS Th17 cells through the production of cytokines like IL-6 and IL-23.

A passive transgenic model of B cell–dependent EAE has been developed involving double-transgenic donor mice. Single transgenic mice in which B cells alone express MHC class II (B^MHC^) do not develop active EAE after either MOG_35–55_ or rrMOG_1–125_ immunization, or after passive EAE induced with pathogenic CD4 T cells from MOG_35–55_–immunized mice.^66^ B cells can support limited CD4 T cell activation, as CFSE-labeled CD4 T cells from 2D2 mice proliferated more in MOG_35–55_–challenged B^MHC^ mice than in mice completely lacking MHC class II. In line with this, previously primed 2D2 CD4 T cells produced more IFNγ when cocultured with splenocytes from B^MHC^ mice than when cocultured with splenocytes from MHC class II–deficient mice. Ultimately, both responses were significantly lower than that of WT B6 mice.^66^ B cell–dependent passive EAE was achieved in double-transgenic IgH^MOG^ mice in which B cells are the only cells that express MHC class II. These B^MHC^ × IgH^MOG^ mice do not develop EAE when immunized with rhMOG. However, adoptively transferring CD4 T cells from MOG_35–55_–immunized mice successfully induced disease in these mice.^65^ This model requires both B cell antigen presentation along with an enrichment in rat MOG–specific precursor B cells, as B^MHC^ mice did not develop EAE after receiving CD4 T cells from MOG_35–55_–immunized mice.^65^^,^^66^ WT and double transgenic mice showed more spinal cord inflammation and demyelination than B^MHC^ mice after receiving CD4 T cells from MOG_35–55_–immunized mice. Notably, transfer of MOG-specific Abs followed by rhMOG immunization in B^MHC^ mice does not induce disease, indicating that the disease seen in B^MHC^ × IgH^MOG^ mice requires mechanisms beyond Ab production.^65^ B cells from B^MHC^ × IgH^MOG^ mice induced significantly more proliferation in MOG-specific CD4 T cells from a hybridoma than B cells from B^MHC^ mice, indicating B cells in this double transgenic model enhance antigen-specific CD4 T cell responses.^65^

## Regulatory B cells

A regulatory role for B cells in EAE has also been described. While MOG_35–55_–induced EAE is not B cell dependent, depleting B cells prior to immunization resulted in worse and faster disease onset with more CNS inflammation and demyelination than in B cell–sufficient B6 mice.^57^^,^^70^ These B cell–depleted mice showed an increased number of CD4 T cells secreting IFNγ and IL-17A, suggesting that B cells may act by inhibiting pathogenic CD4 T cell activation/polarization. Specifically, CD1d^hi^CD5^+^ B cells were shown to reduce disease severity when transferred into B cell–depleted mice prior to MOG_35–55_ immunization compared with B cell–depleted mice receiving non-CD1d^hi^CD5^+^ B cells.^21^ IL-10 production by these cells is likely a key driver of their regulatory function, as CD1d^hi^CD5^+^ B cells produced more IL-10 than non-CD1d^hi^CD5^+^ B cells. Bone marrow chimeric mice, in which B cells alone lack the ability to produce IL-35, experienced worse EAE, had more IFNγ- and IL-17A–producing CD4 T cells, and exhibited an increase in CD4 T cells in the CNS compared with control mice following MOG_35–55_ immunization.^22^ Notably, CD138^+^ plasma cells were the major B cell subset producing IL-35. While WT B6 mice will enter a remission phase at later stages of disease, B cell–deficient mice developed severe, nonremitting EAE that fails to resolve.^98^ Late-stage recovery from EAE was shown to depend on the ability of B cells to produce IL-10, as mixed bone marrow chimeras in which B cells alone lacked the ability to produce this cytokine did not go into remission, as did their WT counterparts. It was found that B cells that express CD80 and CD86 are crucial for producing IL-10 to aid this disease recovery, as B7-deficient B cells did not remediate disease.^99^ However, as post–disease onset depletion of B cells reduces disease severity,^70^ the regulatory role of B cells in MOG_35–55_–mediated EAE may be limited to the early phases of disease. The NH-terminus of MBP has also been evaluated for a regulatory B cell role in EAE. Immunization with MBPAc_1–11_ in B10.PL mice induced the same severity and disease onset of EAE in B cell–sufficient and B cell–deficient mice but varied in recovery after peak of disease. B cell–sufficient mice exhibited greater recovery, suggesting a regulatory role for B cells during recovery from EAE in this model.^100^ In BALB/c mice immunized with PLP_180–199_, the incidence and onset of EAE were similar between B cell–sufficient and B cell–deficient groups, but the clinical course was more severe in B cell–deficient groups.^34^ It remains to be seen whether there is an active regulatory B cell role in protein/large peptide-based models that engage a pathogenic B cell response.

## Conclusions

The pathogenic role of B cells in MS is underscored by the success of B cell depletion therapy. Understanding B cell phenotypes, functions, and the cellular interactions in which they participate during MS disease development and progression is of critical importance. This will require a variety of in vivo models to fully uncover their pathogenic mechanisms. The role that B cells play in EAE has been historically understudied, as the majority of EAE models are B cell independent. This review provides a summary of the approaches used to feature pathogenic B cells in EAE. These models have been valuable in identifying B cell antigen presentation, cytokine secretion, and Ab production as important disease-relevant functions, though the necessity of these features varies between models. Active models like rhMOG have a B cell antigen presentation component and also rely on pathogenic Abs. B^MHC^ × IgH^MOG^ transgenic mice that develop passive EAE display B cell antigen presentation–dependent disease. Various spontaneous models of EAE driven by B and T cell transgenics are powerful tools to study pathogenic B cell biology. Finally, immunizing WT B6 mice with PLP_ECD_ represents a novel complimentary B cell–dependent model with the interesting caveat that it is antigen presentation dependent but independent of pathogenic Ab production.

Approximately 85% of human patients present with relapsing-remitting MS (RRMS).^101^ SJL mice have been a powerful tool to model multiphasic, RRMS-like disease.^75^ There are many studies documenting relapsing disease with PLP_139–151_ and PLP_178–191_ in SJL mice,^25^^,^^72^^,^^102^ though a significant role for B cells in these contexts has not been reported. Epitope spreading is thought to be a primary mechanism driving disease relapse in both adult^103^ and pediatric^104^ MS, which has also been demonstrated in SJL mice.^25^ Given the success of BCDT in RRMS patients, it is likely B cells play a role in supporting relapses. Thus, developing B cell–dependent relapsing EAE models may be an interesting approach in mimicking human disease.

While the previously described models address how myelin peptides, antimyelin Abs, and genetic predisposition influence the role of B cells in disease development, little is known about how infections contribute to this process. EBV has been identified as a potential trigger for MS.^5^^,^^105^ Notably, EBV establishes latency in B cells, potentially linking pathogenic B cells to viral infection. It has been shown that murine gamma herpes virus 68, the murine homolog to EBV, exacerbates EAE in MOG_35–55_–immunized animals,^106^ and a recent report demonstrated the same in an EBV-infected humanized mouse EAE model.^107^ Vaccination targeting EBV is being explored to potentially prevent MS.^108^ B cell–dependent models will certainly be of value in understanding virus–B cell dynamics in CNS-demyelinating disease etiology.

Ultimately, specific targeting of pathogenic B cells may lead to better therapeutics while sparing those with regulatory potential, and may minimize the risk of immunosuppression associated with anti-CD20 therapies.^109^ Collectively, animal models of MS that feature pathogenic B cells continue to be increasingly important as the field interrogates translatable immunobiology for deeper understanding of neuroinflammatory CNS demyelinating disease.

## Data Availability

No new data were generated for this work.
